# Inversion of force lines in fiber-reinforced jammed granular material

**DOI:** 10.1140/epje/s10189-021-00053-6

**Published:** 2021-04-24

**Authors:** Pavel S. Iliev, Falk K. Wittel, Hans J. Herrmann

**Affiliations:** 1 ETH Zurich, Computational Physics for Engineering Materials, Institute for Building Materials, Stefano-Franscini-Platz 3, 8093 Zurich, Switzerland; 2 ETH Zurich, Complex Materials and Systems, Institute for Building Materials, Stefano-Franscini-Platz 3, 8093 Zurich, Switzerland; 3 PMMH, ESPCI Paris, 7 quai St. Bernard, 75005 Paris, France; 4 Departamento de Física, Universidade do Ceará, Fortaleza, 60451-970 Brazil

## Abstract

**Abstract:**

Freestanding columns, built out of nothing but loose gravel and continuous strings can be stable even at several meters in height and withstand vertical loads high enough to severely fragment grains of the column core. We explain this counter-intuitive behavior through dynamic simulations with polyhedral rigid particles and elastic wire chains. We evaluate the fine structure of the particle contact networks, as well as confining forces and reveal fundamental intrinsic differences to the well-studied case of confining silos.

**Graphic abstract:**

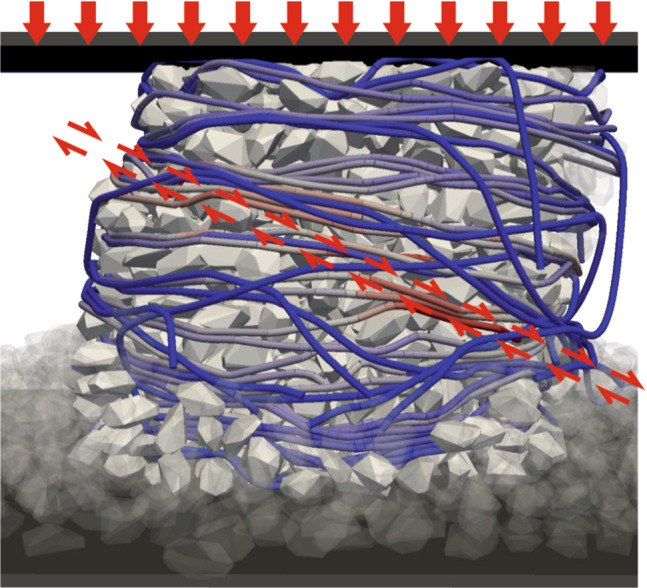

## Introduction

**Fig. 1:**
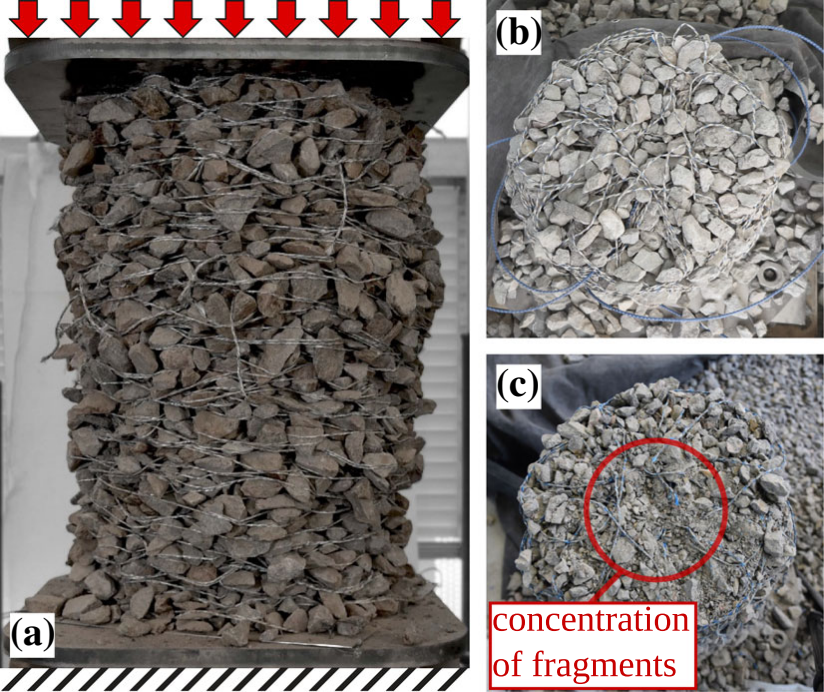
Freestanding experimental jammed wire-reinforced granular column with initial diameter 330 mm before loading. **a** The state of the material inside a column after loading with a force $F_{z}=60kN$ **b** and $F_{z}=200kN$ **c**

Granular materials in the form of freestanding structures in the absence of additional confinement can be realized through so-called designed granular matter [1–3], consisting of pre-fabricated particles of particular shape. These particular methods are based on the non-convexity of the designed particles, utilizing their possibility to interlock. This approach gave rise to a new paradigm for fabricating jammed granular materials composed of initially disordered particles that can form stable structures when being packed. It is an interesting question if also ordinary disordered granular materials can become freestanding and load-bearing. Aejmelaeus-Lindström *et al.* [4] showed that through the use of a thread or a wire, carefully placed inside gravel particles in a layer-wise manner, jamming can be induced. Both approaches have in common the aim of transforming an initially disordered granular material to a solid-like jammed material. However, the latter approach makes use of a very peculiar form of confinement. Since decades, reinforcement through fibers or wires has been used in soils for geotechnical applications to increase the cohesion and strength of the material [5–10], but in the combination proposed by Aejmelaeus-Lindström et al., the wire has also a confining role. Further experimental investigations were carried out on the wire-reinforced jammed architectural structures by Rusenova et al. [11], where large scale columns were tested under uniaxial unconfined compression. In these experiments, fragmented particles were found along the central axis of the sample, suggesting the presence of strong vertical force chains at the center of the granular column (see Fig. 1).

Several key observations regarding the mechanical behavior of jammed wire-reinforced granular structures have been made. For instance, the approximately linear pre-failure stress–strain response, the high-strength under vertical loads, and the accumulation of fragmented particles and broken wire segments located along the column centers as shown in Ref. [11] and in Fig. 1. Moreover, by means of parametric studies performed with numerical methods [12], conditions beneficial to the stability of the columns were identified. However, none of these studies focuses on the intrinsic mechanism preventing the collapse of the structures or investigates the force redistribution caused by the interaction between the granular particles and the elastic wire.

Although several numerical [12–15] and experimental [11, 16, 17] studies have been performed on granular matter reinforced with continuous long wires and key parameter dependencies on the stability as well as the behavior of the structures have been identified, a deeper understanding of the underlying mechanisms allowing for the material to jam under low and high compressive loads is still lacking. Therefore, two fundamental questions remain: “How is structural collapse prevented?” and “How does the interaction between the granular particles and the elastic wire affect the load redistribution in different loading conditions?”. To answer those questions, we employ in this work our numerical framework developed previously and described in detail in Ref. [12, 18]. We show the formation of dominant force-chains located near the column centers during uniaxial loading. Furthermore, by investigating the fabric tensor field, we look into the structure of the contact network and quantify the anisotropy of the structural backbone of the columns. We find substantial differences with the contact force networks in granular columns inside a rigid cylindrical confinement. The principal fabric and stress tensors are further investigated and an inversion of the principal orientations is observed. Recently, Mahajan *et al.* in Ref. [19] observed a similar effect, manifested in a deviation from Janssen’s law [20] for narrow granular columns by investigating the ratio between the apparent mass and added mass together with its dependence on the filling height.

**Fig. 2:**
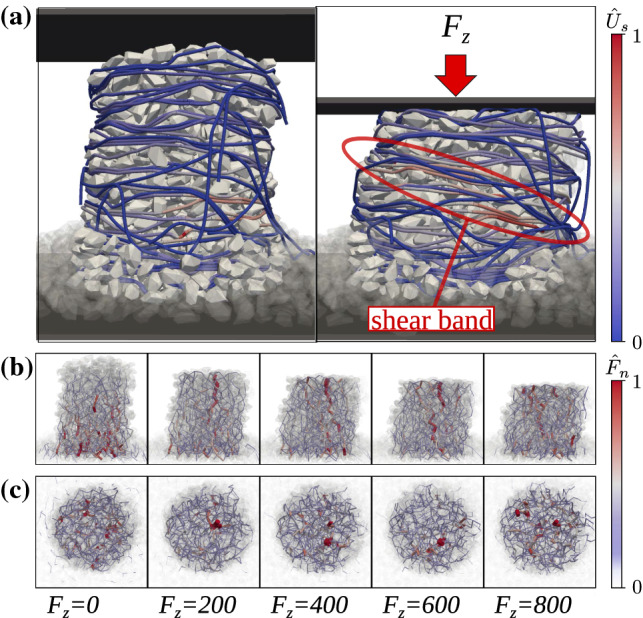
**a** Freestanding numerical jammed wire-reinforced granular column with $N_{l}^{w}=25$ before loading (left) and after loading (right). The inter-particle force network at different loads viewed from the side (**b**) and from the top (**c**). Colors on the wire represent the normalized elastic strain energy $\hat{U}_{s} = U_{s}/max(U_{s})$ for each wire element and colors as well as the thickness of the lines on the force networks represent the normalized force magnitude $\hat{F}_{n} = F_{n}/max(F_{n})$ between each contacting pair of particles

## Numerical method

At the core of the numerical simulations are randomly shaped convex polyhedral particles with their interactions. We employ the Non-Smooth Contact Dynamics (NSCD) method [21, 22], originally proposed by Moreau [23]. This method is well-suited for modeling dense packings of rigid, frictional particles with lasting contacts. Recent studies [24–26] also show the efficiency of this method for modeling polyhedral particles. The NSCD method is based on volume exclusion constraint and Coulomb’s friction law. Because of the discontinuous nature of the contact laws, an implicit integration of the equations of motion:

1 $$\begin{aligned} \begin{aligned} m_{i} \frac{d}{dt} \mathbf {v}_{i}&= \mathbf {F}_{i} + \mathbf {F}^{ext}_{i} , \\ \underline{\mathbf {I}}_{i} \frac{d}{dt} \varvec{\omega }_{i}&= \mathbf {T}_{i} + \mathbf {T}_{i}^{ext} , \end{aligned} \end{aligned}$$

is required for each particle *i*. Where $m_{i}$ and $\underline{\mathbf {I}}_{i}$ are the mass and moment of inertia tensor of particle *i*, $\mathbf {v}_{i}$ and $\varvec{\omega }_{i}$ are its translational and rotational velocities, $\mathbf {F}_{i}$ and $\mathbf {T}_{i}$ are forces and torques from contacts, while $\mathbf {F}^{ext}_{i}$ and $\mathbf {T}^{ext}_{i}$ are external forces and torques. The contact forces $\mathbf {F} = F_{n} \mathbf {n} + \mathbf {F}_{t}$ between the particles are calculated with an iterative scheme based on a Gauss–Seidel algorithm for each time step until a desired global convergence criterion is satisfied. The validity of the Coulomb condition $F_{t} \le \mu ^{p} \Vert \mathbf {F}_{n} \Vert$ is checked for each existing contact to calculate the friction forces.

The elastic wire is modeled as a chain of point-like masses connected by tensile spring elements and rotational springs attached to each node [13, 27]. The tensile force for a wire element is ${f}^{elo} = k^{elo} \epsilon ^{elem}$ with the elongational elastic constant $k^{elo}$ and the element strain denoted by $\epsilon ^{elem}$. The bending moment for a wire node is $M^{rot} = k^{rot} \nu ^{node}$ with the rotational elastic constant $k^{rot}$ and the node angle $\nu ^{node}$ given by the angle between the two elements connected at the node. Self-interaction of the wire is realized with the soft particle (SP) discrete element method [28] introduced by Cundall and Strack [29] with a linear spring-dashpot model for simplicity. Each wire element consists of a spherocylinder [30] with radius $r^{w}$ to carry out the overlap computation (see Ref. [31]). The forces of a contact between two elements are distributed to each of the two nodes for the respective elements with weights inversely proportional to their distance from the closest point of contact [31]. A $5^{th}$ order Gear predictor-corrector method is used to integrate the equations of motion (Eq. 2) for the translational degrees of freedom of each node $n_{j}$:

2 $$\begin{aligned} \begin{aligned} m_{j} \frac{d^{2}}{dt^{2}} \mathbf {r}_{j}&= \mathbf {f}_{j} + \mathbf {f}^{el}_{j} + \mathbf {f}^{ext}_{j}, \end{aligned} \end{aligned}$$

where $m_{j}$ is the mass of node *j*, $\mathbf {r}_{j}$ denotes the position vector, $\mathbf {f}_{j}$ is the contact force, $\mathbf {f}^{el}_{j}$ is the elastic force due to stretching and bending and $\mathbf {f}^{ext}_{j}$ denotes external force on the wire. The contact force $\mathbf {f}$ consists of normal and tangential components $f_{n} = k^{w}_{n} \xi _{n} + \gamma ^{w}_{n} \dot{\xi _{n}}$ and $\mathbf {f}_{t} = k^{w}_{t} \mathbf {\xi }_{t} + \gamma ^{w}_{t} \dot{\mathbf {\xi }_{t}}$ , where $k^{w}_{n}$ and $k^{w}_{t}$ are the elastic constants, $\gamma ^{w}_{n}$ and $\gamma ^{w}_{t}$ are the damping coefficients for the normal and tangential interactions, respectively, and with $\xi _{n}$ and $\mathbf {\xi _{t}}$ we denote the indentation depth and the tangential displacement. Dynamic friction is taken into account by removing the spring when the condition $f_{t} \le \mu _{s}^{w} \Vert \mathbf {f}_{n} \Vert$ is violated and replacing the tangential force by $\mathbf {f}_{t} = \mu _{d}^{w} f_{n} \mathbf {f}_{t}/ \Vert \mathbf {f}_{t} \Vert$, where $\mu _{s}^{w}$ and $\mu _{d}^{w}$ are the static and dynamic friction coefficients of the wire. The contributions of the elastic forces due to elongation and bending of the wire are stored in the vector $\mathbf {f}^{el}$.

Similar to the spherocylinders of the wire segments, the polyhedra are dilated with a spherical particle of radius $r^{p}$, which in our simulations is set to be equal to the wire radius $r^{w}$. This spherical dilatation of polyhedra is known as spheropolyhedra and it is a well-established technique for SP discrete element simulations of irregular particles [32–34]. Note that for the particle–particle interaction, we also consider spheropolyhedra, instead of the sharp-edged polyhedra, which does not change the contact law or the contact calculation procedure. Since the NSCD method is implicit and the SP method is explicit, two different time steps $\Delta t^{NSCD}$ and $\Delta t^{SP}$ are used and a sub-cycling procedure is implemented to transfer forces and torques between particles and wire. A detailed description of the hybrid model and the coupling scheme can be found in Refs. [12, 18]. Similarly to previous numerical studies, the simulation units are non-dimensional as the NSCD is typically employed in a dimensionless form.

The numerical procedure for generating stable, freestanding, wire-reinforced granular columns is the same as in Ref. [12]. Particles and wire are sequentially deposited under gravity in a layer-wise manner inside a rectangular container with a frictional bottom wall and frictionless sidewalls. A single layer of particle constitutes of randomly generated particles placed on two staggered regular lattices. In between two particle layers a double wire loop with disconnected ends is placed. After the system is relaxed, the walls are removed slowly and a stable column remains. The radius of the stable column after the wall removal corresponds to the radius of the wire loop. After a certain simulation time and when the kinetic energy is nearly zero, a vertical load $F_{z}$ is applied to the column through a horizontal top plate, constrained from rotations. The force $F_{z}$ is linearly increased, meaning a constant loading rate. The gravity is kept throughout the wall removal and the loading procedure. For the cylindrical system particles are deposited under gravity and the friction coefficient $\mu ^{w}$ with the walls is $\mu ^{w} = \mu ^{p}$.

Due to the approximate rotational symmetry of the system, averaging around the central axis is performed on all measured variables to reduce the dimensionality of the results [35]. This procedure is essentially a mapping to cylindrical coordinates and averaging around the polar angle resulting in a two-dimensional representation.

## Results

We study here wire-reinforced granular columns with radius approximately 6 times the radius of the biggest particles, with number of wire layers $$N_{l}^{w} =$$ 20, 25, and 30. An increase in number of layers leads to an increase in column height and number of particles in the system. Note that the ratio between cylinder diameter and particle size along other parameters can significantly affect the load distribution for the silo case and lead to counter-intuitive results in the extreme case of a very narrow cylinder [19]. Therefore, we only focus here on the comparison between wire-reinforced and rigid cylinder systems of equivalent sizes and for the considered set of investigated parameters. We are interested in the static case before loading and in the evolution during the quasi-static loading. An example of such a column is shown in Fig. 2a for $$F_{z} = 0$$ (left) and $$F_{z} = 800$$ (right). For both cases, larger stresses are found near the center of the column due to the lateral forces, which is evident even when only body forces act on the system. However, we see that due to the shear band formed at a high load, the wire gets stretched along the shearing region (marked in Fig. 2a by a red ellipse), indicated by the high wire strain energy within the failure zone. To get an insight into the load redistribution between the particles, we show a sequence of snapshots of the inter-particle force network at different loads viewed from the side (Fig. 2b) and from the top (Fig. 2c). At the initial configuration when the sample is not subjected to an external load, the force magnitudes increase from top to bottom. When we start loading the column, we see the formation of strong force chains in the vertical direction for loads $$F_{z} = $$ 200, 400, and 600. Despite the small system size, limited by the numerical complexity, it is noticeable that the force chains are located near the column center as observed experimentally in larger columns [11]. At even higher loads ($$F_{z}=800$$), the force chains are no longer aligned in the vertical direction, since they resist not only against the vertical loads but also the shearing forces. Nevertheless, the strong force chains are still confined within the central region of the samples.

**Fig. 3:**
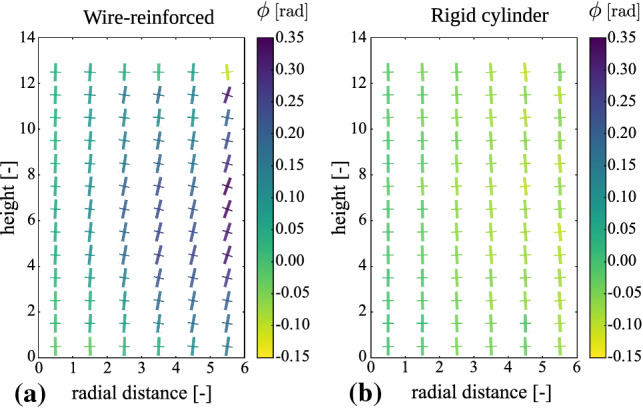
Fabric tensor fields for a wire-reinforced granular column **a** for $N^{w}_l = 25$ and granular material inside a rigid cylinder **b** for $N^{c} = 1300$ expressed in cylindrical coordinates and averaged around the central axis. The largest and smallest principal vectors of the fabric tensors are shown by thick and thin lines, respectively. The magnitudes of the lines represent the principal values of the fabric tensors and the colors represent the signed angle between the vertical axis and the biggest principal vector

Due to the absence of lateral walls, measures used in Ref. [19] cannot be applied to the studied system. For the study of the structural backbone of the system on the micro-level, we calculate the fabric tensor field from the particle-particle contact force network. The fabric tensor is defined as:

3 $$\begin{aligned} \underline{\mathbf {F}}_{i} = \frac{1}{N_{i}^{c}} \sum _{c=1}^{N_{i}^{c}} \mathbf {n}^{(c)} \otimes \mathbf {n}^{(c)}, \end{aligned}$$

where the sum of the dyadic products $\otimes$ of the normal contact vectors $\mathbf {n}^{(c)}$ spans all $N_{i}^{c}$ contacts *c* of the particle *i*. The fabric tensor is a measure of the contact anisotropy and reveals the fine structure of the contact network. Typically, reduced representations of the fabric tensors are used, such as the trace and the deviatoric components or anisotropy coefficients [36–39] to derive conclusions for the anisotropy of granular materials, but these measures do not show the fine details of the contact network. For this reason, the principal vectors of the fabric tensor are calculated and the resulting vector field is shown in Fig. 3a. To compare the wire-reinforced jammed granular column to a system governed by arch formation as a mechanism for load redistribution, we show in Fig. 3b the fabric tensor field of a granular column with no wire of an equivalent size inside a rigid cylinder. It is evident that for both systems close to the central axis, i.e., close to zero in the *x*-axis of Fig. 3, the principal vectors are approximately aligned to the vertical axis. However, at a further distance from the center, the angles formed between the vertical axis and the principal fabric vectors have opposite signs for both systems. This leads to the inversion of the force lines as compared to the well-studied silo system. In the silo case (Fig. 3b), the loads are redistributed outward, toward the cylinder walls, while opposite to that, in Fig. 3a, one observes that the loads are redirected inward, toward the column center. Thus, the confining mechanisms of the rigid cylinder and the elastic wire-reinforcement are fundamentally different in nature. Unlike the case in the cylinder, where due to the rigid confinement arches form within the force network, in the wire-reinforced material, the forces are anchored toward the central axis preventing the collapse of the columns.

**Fig. 4:**
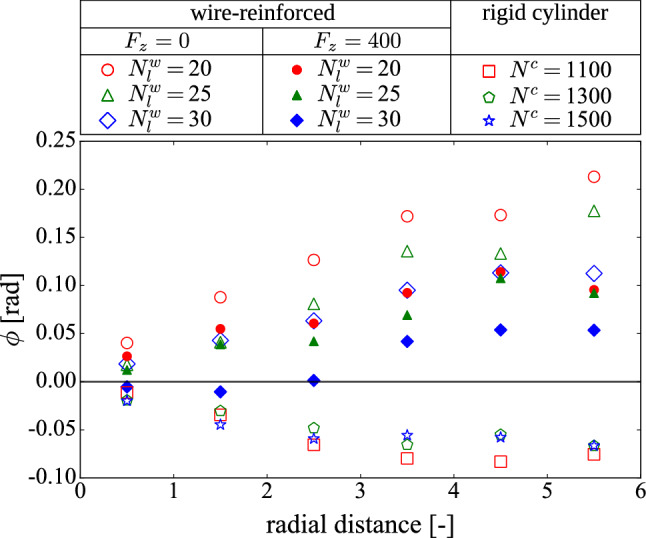
Average principal fabric tensor orientations as a function of the radial distance from the column center for both a wire-reinforced column, denoted by superscript $^w$ and a rigid cylinder, denoted by superscript $^c$. The orientation is given by angle $\phi$ in radians, obtained between the principal direction and the *z*-axis. Positive angle means the principal vector is located in the $I^{st}$ quadrant and negative angle in the $II^{nd}$ quadrant

We further proceed by quantifying the average orientation of the greater principal vector of the fabric tensor by measuring its angle with the vertical axis. The results for both the wire-reinforced and the rigid cylinder systems are shown in Fig. 4 as a function of the radial distance. For the wire-reinforced case, we see that the average angles are always positive, even when a load is applied, although the effect is less predominant for higher columns. On the other hand, for the confining cylinder, the average angles are negative at any distance from the column center. This result further indicates to intrinsic differences between the silo system and the novel wire-reinforced structures for the studied parameters and system sizes.

**Fig. 5:**
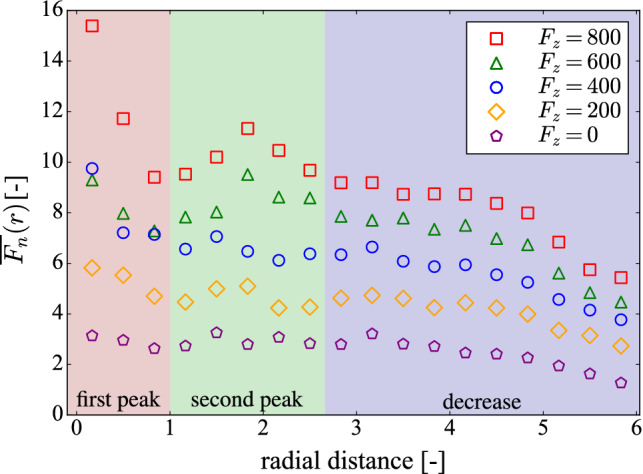
Average inter-particle normal contact force $\overline{F_{n}}(r)$ of the wire-reinforced column for $N_{l}^{w} = 25$ as a function of the radial distance for different loads

Since the fabric tensor is a measure of the anisotropy of the contact network, one expects to see the anchoring effect, leading to larger forces toward the center of the column. Indeed, this becomes visible, when the average magnitude of the particle-particle contact forces $\overline{F_{n}}(r)$ is plotted as a function of the radial distance (see Fig. 5). In the initial configuration, before the loading begins, we see no noticeable maximum, which indicates an approximately homogeneous radial dependency of contact forces. However, when the column is being loaded, there is a peak at the proximity of the center. As expected, there is an increase in all values along the radial direction, but a more unexpected observation is the existence of a small second maximum, which is noticeable at all loads. This second peak in the distribution is due to the branching of the large force chains as can also be observed in Fig. 2b, c. One can see that there is a characteristic distance between the second peak and the column center, related to the system dimensions. These phenomena, resulting in the concentration of large forces could explain the congregation of broken particles and wire segments, observed previously in Ref. [11] and also discussed in Ref. [18].

**Fig. 6:**
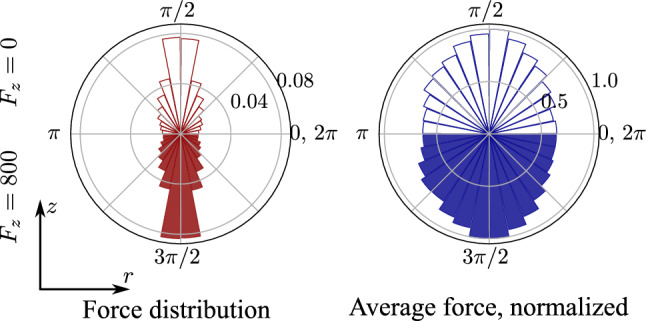
Polar histogram of the contact orientation (left) and normalized polar average of the contact force (right) for the contacts between the wire and the particles for $N_{l}^{w} = 25$ and $N^{c} = 1300$ as function of the angle $\theta$ (in radians) between the force vectors and the *z*-axis with no load applied (top) and with applied vertical load $F_{z}=800$ (bottom). The distribution on the right is normalized with respect to the largest average force in order to be able to compare both cases. The *z*-axis is aligned with the $90^{\circ }$ orientation. Note that for both polar distributions, the condition $p(\theta ) = p(\theta + \pi )$ is valid, therefore, it is sufficient to analyze only two quadrants

In order to explain in more detail the microscopic behavior, one needs to also investigate the local interaction between the wire and the particles. This can be done by looking at the orientation of particle-wire contact forces. Figure 6 shows the distribution of force orientations (left) and the average force magnitudes in polar plots. The distributions are obtained by taking into account only the force vector components in the $\mathbf {z}-\mathbf {r}$ plane, where $\mathbf {z}$ is the unit vector in the vertical direction, and $\mathbf {r}$ is the radial unit vector of the contact point. Thus, the plots represent the ratio between vertical and lateral contact forces between the particles and the wire. Interestingly, the majority of the forces are oriented in the vertical direction, meaning that wire segments are activated by being squeezed between particles. The lateral forces counteracting the outward flow of particles are therefore transmitted to the wire by tension forces, instead of the wire directly balancing those lateral forces. This is further observed in the right side of Fig. 6, where one sees again an anisotropic distribution with the largest forces being in the direction of the *z*-axis. Moreover, at high loads (bottom parts), even though the average force distribution becomes slightly wider and tends toward a more isotropic distribution, the structure is not generally changed. This widening of the average force distribution in the radial direction is likely caused by the shear band and the increased lateral forces at certain regions of the structure. Thus, we demonstrate that the confining function of the wire-reinforcement is mostly in the redistribution of loads in the inter-particle force network rather than in the supporting and force-balancing of the structure.

## Conclusions

We have numerically investigated the microscopic effects preventing wire-reinforced jammed granular structures from collapsing and allowing them to support mechanical loads using a hybrid DEM model that has been previously developed. Characteristic features observed experimentally [11], such as shear band formation and concentration of strong force chains near the vicinity of the column center were qualitatively reproduced and reported.

It is of main interest to understand the structural backbone of the granular material not only during loading but also with only gravitational forces acting on the structure, by calculating and analyzing the fabric tensor field. In contrast to an equivalent system confined by a rigid cylinder, the principal vectors of the fabric tensor for the wire-reinforced material are oriented toward the column center instead of pointing outward toward the cylinder walls. From this result, we conclude that the load redistribution is fundamentally different for the two systems and for the latter we explain this observation by an anchoring effect of the wire, acting as intrinsic confinement. Additionally, we suggest that a study on the dependence of the height/radius ratio of the fabric tensor field could further extend the understanding of the effect reported by Mahajan et al. [19].

Furthermore, the radial dependence of the average inter-particle normal contact force magnitude confirmed the existence of a peak at the center column when a vertical load is applied to the structure. Interestingly, a small second peak in the distribution is also observed for all loading states, caused by the branching of the strongest force chain. Lastly, by looking at the orientation of wire-particle forces, we conclude that most of the wire elements are compressed between granular particles, which as a result activates the tensile forces in the wire. The collective effect of these mechanisms manifests itself in freestanding and even load-bearing granular structures without any cohesive forces.
